# Hospitalization for acute coronary syndrome increases the long-term risk of pneumonia: a population-based cohort study

**DOI:** 10.1038/s41598-021-89038-1

**Published:** 2021-05-06

**Authors:** Joonghee Kim, Sang Jun Park, Sangbum Choi, Won-woo Seo, Yeon Joo Lee

**Affiliations:** 1 Department of Emergency Medicine, Seoul National University Bundang Hospital, Bundang-gu, Seongnam-si, Gyeonggi-do Republic of Korea; 2 Department of Ophthalmology, Seoul National University Bundang Hospital, Bundang-gu, Seongnam-si, Gyeonggi-do Republic of Korea; 3 Department of Statistics, Korea University, Seoul, Republic of Korea; 4 Division of Cardiology, Department of Internal Medicine, Kangdong Sacred Heart Hospital, Hallym University College of Medicine, Seoul, Republic of Korea; 5 Division of Pulmonary and Critical Care Medicine, Department of Internal Medicine, Seoul National University Bundang Hospital, 82, Gumi-ro 173Beon-gil, Bundang-gu, Seongnam-si, Gyeonggi-do 13620 Republic of Korea

**Keywords:** Cardiology, Risk factors, Epidemiology

## Abstract

It is well established that the risk of acute coronary syndrome (ACS) increases after respiratory infection. However, the reverse association has not been evaluated. We tested the hypothesis that the long-term risk of pneumonia is increased after a new ACS event. A matched-cohort study was conducted using a nationally representative dataset. We identified patients with admission for ACS between 2004 and 2014, without a previous history of ACS or pneumonia. Incidence density sampling was used to match patients, on the basis of age and sex, to 3 controls who were also free from both ACS and pneumonia. We examined the incidence of pneumonia after ACS until the end of the cohort observation (Dec 31, 2014). The analysis cohort consisted of 5469 ACS cases and 16,392 controls (median age, 64 years; 68.3% men). The incidence rate ratios of the first and the total pneumonia episodes in the ACS group relative to the control group was 1.25 (95% confidence interval [CI], 1.11–1.41) and 1.23(95% CI 1.11–1.36), respectively. A significant ACS-related increase in the incidence of pneumonia was observed in the Cox-regression, shared frailty, and joint frailty model analyses, with hazard ratios of 1.25 (95% CI 1.09–1.42), 1.35 (95% CI 1.15–1.58), and 1.24 (95% CI 1.10–1.39), respectively. In this population-based cohort of patients who were initially free from both ACS and pneumonia, we found that hospitalization for ACS substantially increased the long term risk of pneumonia. This should be considered when formulating post-discharge care plans and preventive vaccination strategies in patients with ACS.

## Introduction

Ischemic heart disease (IHD) and pneumonia are major causes of morbidity and mortality worldwide^1,2^. The American Heart Association reported that 15.5 million persons (≥ 20 years of age) in the US have IHD^3^, and although the mortality rates of IHD are decreasing, it still is the leading cause of death^3–5^. Meanwhile, pneumonia is the most common cause of adult hospital admissions. Approximately 1 million adults in the US are hospitalized with pneumonia every year, and approximately 50,000 die from this disease^6,7^. Likewise, these two diseases are associated with a significant social burden in terms of healthcare resource utilization and social economic cost^8^.

An association between pneumonia and the occurrence of coronary artery disease (CAD) in the days and weeks after a respiratory infection has been well established^9–12^. Although there have been conflicting results regarding the long-term risk of CAD after pneumonia^13–15^, Corrales-Medina at el. recently demonstrated that hospitalization for pneumonia was associated with an increased long-term risk of CAD, for up to 10 years after the respiratory infection^16^. The pathophysiology is thought to be as follows. Systemic and coronary artery inflammation increases cardiovascular risk^17–19^; infection promotes platelet activation^20,21^ and thrombosis^22^; changes in nitric oxide (NO) synthase and cyclooxygenase (COX) lead to endothelial dysfunction^23,24^; pneumonia leads to changes in myocardial contractility, oxygen demand, and delivery^25–27^; and the microorganism can have a direct effect on cardiovascular risk^28,29^.

However, most of the aforementioned mechanisms (increased proinflammatory cytokines, prothrombotic activation, and endothelial dysfunction) are also observed in patients with IHD, especially in patients with acute coronary syndrome (ACS). Thus, these findings may also affect pneumonia development after ACS, reflecting a reverse association. Therefore, we tested the hypothesis that the risk of pneumonia development is increased after an ACS event. In the present study, we assembled a population-based cohort of patients with ACS and age-sex matched controls without ACS to verify the impact of a new ACS event on the incidence of pneumonia for up to 10 years.

## Methods

### Study design

A matched-cohort study was conducted using a nationally representative dataset. The patients admitted for ACS without prior history were included as study group (ACS group). Three controls for each of the patient were randomly selected using incidence density sampling. We examined the risk of pneumonia up to 10 years using the parallel cohorts. This study was conducted in accordance with the amended Declaration of Helsinki. The institutional review board(IRB)s of the study site (Seoul National University Bundang Hospital) approved the study and provided a waiver of informed consent due to the retrospective nature of the study. (IRB number: X-1904–534-902).

### Data source

The data source was National Health Insurance Service—National Health Screening Cohort (NHIS-HEALS, 2002–2015), a population-based cohort of national health screening enrollees recruited at 2002 and 2003 (10% of 5,150,000 enrollees ranged in age from 40 to 79 years in December 2003) and observed from 2002 to 2015^30^. The dataset includes the results of various health screening programs as well as the whole claim information of the enrollees including diagnostic codes, prescription, and procedure codes and related costs from 2002 to 2015. The diagnostic codes follow the 6^th^-revision of Korean Classification of Diseases, which was developed based on the 10th-revision of International Classification of Diseases (ICD) coding system. The dataset also includes information about disability and death based on the national disability registration data and death certificates, respectively.

### Definition of the exposure and outcome events

The ACS cohort comprised adult patients (aged 20 years or older) admitted with a primary diagnosis of ACS (I21.x, I22.x, I24.x, or I20.0x). The primary outcome was pneumonia or death by pneumonia. An episode of pneumonia was defined by outpatient visit or inpatient care with a primary diagnosis of pneumonia (J10.0x, J11.0x, J12.x, J13.x, J14.x, J15.x, J16.x, J17.x, J18.x) accompanied by a prescription for systemic antibiotics (ATC J01x). Because such events can occur multiple times during a single pneumonia episode, we gathered data on temporally clustered claims of both outpatient visits and inpatient care on the basis of the duration of antibiotic prescription for the former and the length of hospital stay for the latter; when a claim followed within a week of the endpoint of its predecessor, it was considered to be of the same pneumonia episode. After clustering, we selected only those episodes with a total duration of treatment of at least five days. Death by pneumonia was defined as the presence of a death certificate with pneumonia as the cause of death.

### Construction of the study cohort dataset

The time of the study entry was Jan 1, 2004. We built a cohort of patients with and without ACS, using incidence density sampling without replacement. Specifically, we first identified individuals who were admitted for a primary diagnosis of ACS (first admission for each patient). We excluded patients with a previous (from Jan 1, 2002) diagnosis of ACS, primary or secondary, regardless of admission status. We also excluded patients with any previous (from Jan 1, 2002) or concurrent diagnosis of pneumonia, primary or secondary, at the time of admission. Thus, the ACS group comprised patients who were free from both ACS and pneumonia for at least 2 years, from Jan 1, 2002 until the admission. We then matched each member in the ACS group by age (± 1 year) and sex to 3 controls randomly selected from those who were alive during at least the same period, but did not have any diagnosis of ACS or pneumonia (primary or secondary, regardless of admission status) prior to the study entry. Resampling was not allowed, and individuals initially selected as controls could be admitted for ACS at a later time. Covariates including age, sex, smoking status, basal mass index (BMI), diabetes mellitus, hypertension, IHD, stroke, heart failure, chronic renal failure, advanced liver disease, chronic obstructive pulmonary disorder (COPD), and malignancy were based on the most recent health screening report and the claim data of the two-year period before the participant’s inclusion in the study cohort (Additional file 1. Table S1).

### Statistical analyses

Categorical data are reported as frequencies and proportions, whereas continuous data are reported as medians and interquartile ranges (IQRs). Group differences were evaluated using the Wilcoxon’s rank-sum test, chi-square test, or Fisher’s exact test, as appropriate.

Pneumonia can occur multiple times in a patient, which can be understood and analyzed within a recurrent event framework. In addition, death from pneumonia-related conditions works as an informative censoring mechanism, in the sense that death occurs at a higher probability after a pneumonia event, and prevents the further observation of pneumonia in the patient. Informative censoring occurs when patients are lost to follow-up due to reasons related to the study, which is increasingly appreciated as a potential bias affecting the estimation of treatment effects in medical research^31,32^. Therefore, we utilized three modeling approaches to investigate the association between ACS exposure and subsequent pneumonia: (1) a standard Cox-regression model relating ACS exposure to the first event occurrence of pneumonia, (2) a frailty model factoring in the multiple recurrences of pneumonia, and (3) a joint frailty model that simultaneously estimates the ACS effect on multiple recurrences of pneumonia as well as death from pneumonia. Method (1) focuses on the relationship between ACS and the first event of pneumonia, treating death as a non-informative censoring event. Method (2) incorporates random-effects into a Cox-type regression (as shared gamma frailty) to analyze the relationship between ACS and multiple pneumonia recurrences, but is subject to bias since it ignores informative censoring by death. Method (iii) incorporates random-effects into two Cox-type regression models for multiple pneumonia events, as well as death, within a joint modeling framework^33^, and thus, can accommodate the association between pneumonia recurrences and related death, as well as potential bias issues. The proportionality hazard assumption of the standard Cox model was tested using the cox.zph function in R's survival package, in which each of the covariates in the model was tested for a significant interaction with log-transformed time^34^. There was no statistically significant interaction between any of the covariates and time.

*P* values < 0.05 were considered significant. All data handling and statistical analyses were performed using R-packages version 3.3.2 (R Foundation for Statistical Computing, Vienna, Austria). The frailty and joint frailty models, described in methods ii and iii, respectively, were built using the contributed R-package, Frailtypack^35^.

## Results

**Figure 1:**
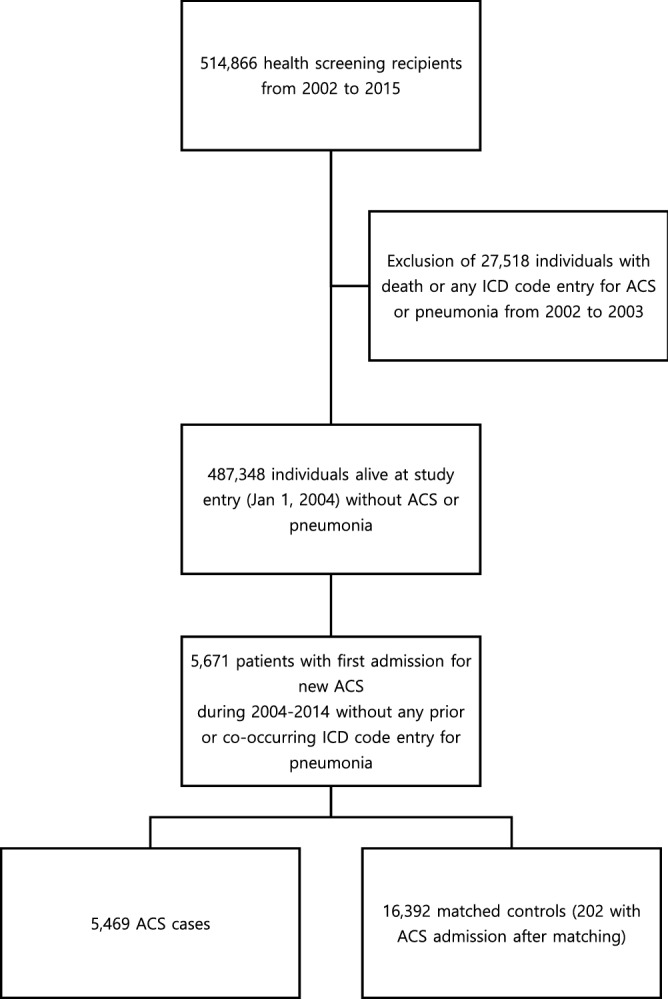
Construction of the study cohort dataset using incidence density sampling.

There were 487,348 eligible individuals in NHIS-HEALS. After matching, the analysis cohort, nested within the NHIS-HEALS, consisted of 21,861 participants (5469 ACS cases and 16,392 controls) (Fig. 1). Of note, 202 controls (1.2%) developed ACS after they were identified as a control using incidence density sampling; however, they were still considered as controls in all analyses. The median age (64 years; IQR, 56–71 years) and the proportion of male patients (68.3%) were same in the exposure and control groups, as expected from the matching process (Table 1). However, BMI, smoking status and most of the comorbidities including diabetes, hypertension, IHD, stroke, heart failure, chronic renal failure, advanced liver disease and COPD were significantly different. The median follow-up duration was shorter in the exposure group (2045 days; IQR, 1053–3145 days) than in the control group (2217 days; IQR, 1244–3243.5 days; *p* < 0.001). This was due to the higher frequency of the censoring event due to death in the exposure group (n = 781, 14.3%) than in the control group (n = 1406, 8.6%, p < 0.001). Despite the shorter observation duration, a total of 361 patients (6.6%) in the exposure group had pneumonia at least once during the follow-up period, while pneumonia occurred in 924 individuals (5.6%) in the control group (*p* = 0.010). The incidence rate ratios (IRRs) of the first and total pneumonia events were 1.25 (1.11–1.41) and 1.23 (1.11–1.36), respectively.

**Table 1:** Baseline characteristics of the study cohort.

	Exposure group	Control group	*p*
	(N = 5469)	(N = 16,392)	
**Age**			0.939
55 or less	1274 (23.3%)	3873 (23.6%)	
56–65	1818 (33.2%)	5444 (33.2%)	
66–75	1714 (31.3%)	5128 (31.3%)	
over 75	663 (12.1%)	1947 (11.9%)	
Median age	64 (56–71)	64 (56–71)	0.560
Sex, male	3735 (68.3%)	11,194 (68.3%)	1.000
**BMI**			< 0.001
Underweight (< 18.5)	96 (1.8%)	432 (2.6%)	
Normal (18.5–22.9)	1461 (26.7%)	5680 (34.7%)	
Overweight (23.0–24.9)	1541 (28.2%)	4485 (27.4%)	
Pre-Obese (25.0–29.9)	2180 (39.9%)	5384 (32.8%)	
Obese (≥ 30)	191 (3.5%)	411 (2.5%)	
Median BMI	24.5 (22.7–26.3%)	23.9 (22.0–25.8%)	
**Smoking**			< 0.001
Non-smoker	3080 (56.3%)	10,087 (61.5%)	
Active smoker	1624 (29.7%)	3678 (22.4%)	
Ex-smoker	765 (14.0%)	2627 (16.0%)	
Diabetes	1210 (22.1%)	2098 (12.8%)	< 0.001
Hypertension	2969 (54.3%)	6095 (37.2%)	< 0.001
Ischemic heart disease	1371 (25.1%)	927 (5.7%)	< 0.001
Stroke	399 (7.3%)	765 (4.7%)	< 0.001
Heart failure	397 (7.3%)	468 (2.9%)	< 0.001
Chronic renal failure	99 (1.8%)	90 (0.5%)	< 0.001
Advanced liver disease	41 (0.7%)	181 (1.1%)	0.029
COPD	207 (3.8%)	417 (2.5%)	< 0.001
Malignancy	281 (5.1%)	883 (5.4%)	0.500
Median follow-up duration (days, IQR)	2045.0 (1053.0–3145.0)	2217.0 (1244.0–3243.5)	< 0.001
Censoring by death	781 (14.3%)	1406 (8.6%)	< 0.001
Pneumonia (N. of patients)	361 (6.6%)	924 (5.6%)	0.010
Pneumonia (N. of total incidents)	510	1330	
Total person years at risk	30,799.8	98,723.3	
Incidence rate ratio (First incidents)	1.25 (1.11–1.41)	Reference	< 0.001
Incidence rate ratio (Total incidents)	1.23 (1.11–1.36)	Reference	< 0.001

BMI: Basal mass index; COPD: Chronic obstructive pulmonary disease.

**Figure 2:**
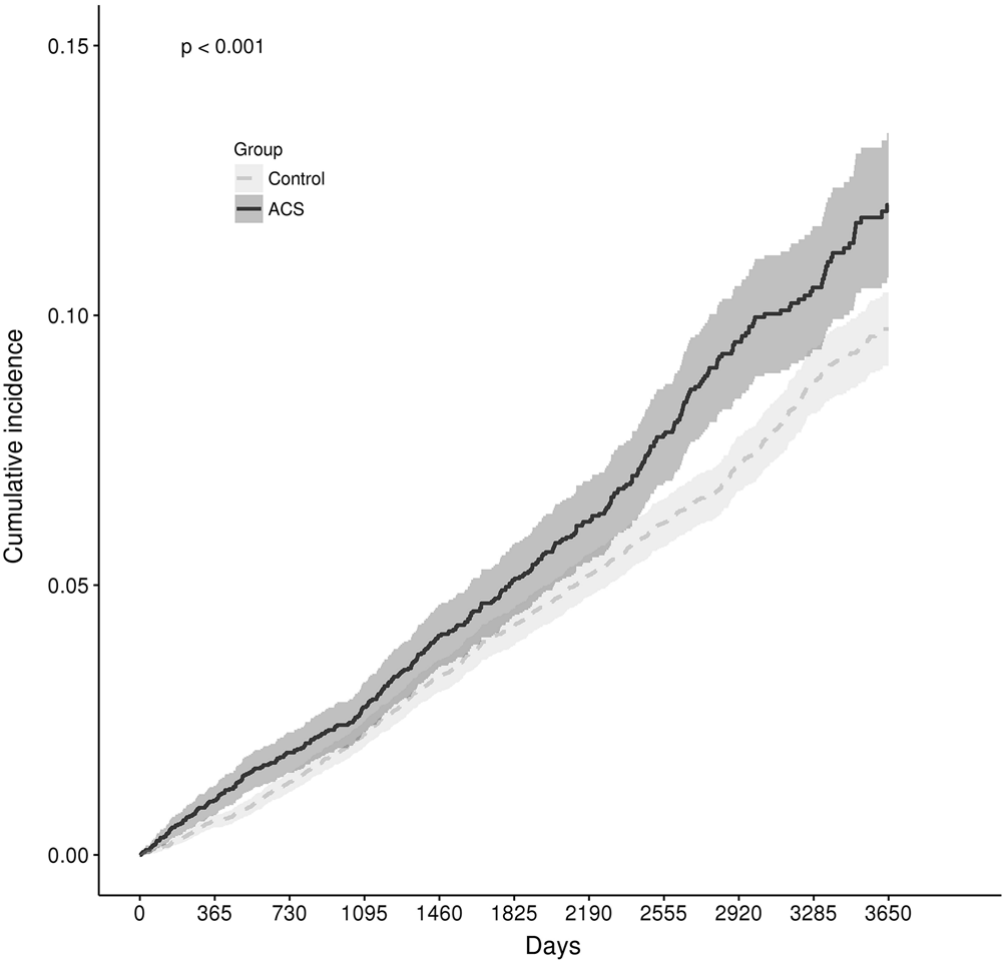
Cumulative incidence of first pneumonia events during the first 10 years after study inclusion. Exposure to ACS was associated with a significantly higher incidence of first pneumonia events during the observation period (log-rank test, *p* < 0001).

The cumulative incidence of the first pneumonia event during the first ten years was compared between the exposure and control groups using the log-rank test (Fig. 2). The incidence of pneumonia was significantly higher in the exposure group than in the control group (*p* < 0.001). The results of the standard Cox-regression modelling analysis of the association between ACS exposure and the risk of pneumonia are shown in Table 2. Exposure to ACS was a significant risk factor for the first incidence of pneumonia (HR, 1.25; 95% CI 1.09–1.42; *p* = 0.001); other risk factors included increased age (HR: 1.77, 95% CI 1.44–2.17 for age 56 to 65 years; HR 3.55, 95% CI 2.92–4.32 for age 66 to 75 years; HR 6.50, 95% CI 5.23–8.08 for age over 75 years; all relative to age 55 years or less), male sex (HR 1.17, 95% CI 1.02–1.33), BMI (HR 1.49, 95% CI 1.14–1.95 for age underweight; HR 0.81, 95% CI 0.70–0.93 for overweight; HR 0.76, 95% CI 0.67–0.88 for pre-obese; all relative to normal BMI), diabetes (HR: 1.22, 95% CI 1.06–1.41), stroke (HR: 1.27, 95% CI 1.04–1.56), heart failure (HR: 1.39, 95% CI 1.11–1.75) and COPD (HR: 2.37, 95% CI 1.93–2.90).

**Table 2:** Standard Cox regression model of the first pneumonia event.

		HR (95% CI)	*P*
Acute coronary syndrome		1.25 (1.09–1.42)	0.001
Age	55 or less	Reference	
	56–65	1.77 (1.44–2.17)	< 0.001
	66–75	3.55 (2.92–4.32)	< 0.001
	over 75	6.50 (5.23–8.08)	< 0.001
Sex, male		1.17 (1.02–1.33)	0.020
Smoking	Non-smoker	Reference	
	Active smoker	1.13 (0.98–1.31)	0.105
	Ex-smoker	0.84 (0.69–1.03)	0.087
BMI	Underweight (< 18.5)	1.49 (1.14–1.95)	0.003
	Normal (18.5–22.9)	Reference	
	Overweight (23.0–24.9)	0.81 (0.70–0.93)	0.003
	Pre-Obese (25.0–29.9)	0.76 (0.67–0.88)	< 0.001
	Obese (≥ 30)	0.77 (0.53–1.10)	0.148
Diabetes		1.22 (1.06–1.41)	0.007
Hypertension		0.94 (0.83–1.06)	0.292
Ischemic heart disease		1.04 (0.87–1.24)	0.657
Stroke		1.27 (1.04–1.56)	0.017
Heart failure		1.39 (1.11–1.75)	0.004
Chronic renal failure		1.17 (0.67–2.03)	0.584
Advanced liver disease		1.01 (0.57–1.78)	0.983
COPD		2.37 (1.93–2.90)	< 0.001
Malignancy		1.05 (0.83–1.32)	0.701

BMI: Basal mass index; COPD: Chronic obstructive pulmonary disease.

Table 3 presents the results of the two recurrent event analyses: (1) the shared frailty model, and (2) the joint frailty model. ACS was associated with a significantly increased risk of pneumonia in both models (HR 1.35, 95% CI 1.15–1.58, *p* < 0.001; and HR 1.24, 95% CI 1.10–1.39, *p* < 0.001, respectively). Other variables significantly associated with an increased risk of pneumonia in the models were age, sex, smoking, BMI, stroke, heart failure and COPD. The results of the sub-model for the terminal event (death) in the joint frailty model are shown in Additional file 2 (Table S2).

**Table 3:** Frailty and joint frailty model of recurrent pneumonia event.

		Recurrent events		Recurrent events with terminal event	
		(Frailty model)		(Joint frailty model)	
		HR (95% CI)	P	HR (95% CI)	P
Acute coronary syndrome		1.35 (1.15–1.58)	< 0.001	1.24 (1.10–1.39)	< 0.001
Age	55 or less	Reference		Reference	
	56–65	2.91 (2.25–3.76)	< 0.001	2.09 (1.74–2.50)	< 0.001
	66–75	5.28 (4.09–6.80)	< 0.001	3.65 (3.06–4.35)	< 0.001
	over 75	15.52 (11.72–20.56)	< 0.001	7.83 (6.45–9.52)	< 0.001
Sex, male		1.38 (1.17–1.63)	< 0.001	1.36 (1.21–1.53)	< 0.001
Smoking	Non-smoker	Reference		Reference	
	Active smoker	1.20 (1.01–1.43)	0.043	1.13 (0.99–1.28)	0.060
	Ex-smoker	0.79 (0.63–0.99)	0.042	0.85 (0.72–1.01)	0.071
BMI	Underweight (< 18.5)	1.59 (1.10–2.31)	0.014	1.42 (1.12–1.80)	0.004
	Normal (18.5–22.9)	Reference		Reference	
	Overweight (23.0–24.9)	0.72 (0.61–0.86)	< 0.001	0.77 (0.68–0.88)	< 0.001
	Pre-Obese (25.0–29.9)	0.77 (0.65–0.91)	0.002	0.80 (0.71–0.90)	< 0.001
	Obese (≥ 30)	0.78 (0.50–1.20)	0.256	0.78 (0.56–1.08)	0- + .133
Diabetes		1.27 (1.06–1.52)	0.011	1.20 (1.05–1.37)	0.006
Hypertension		0.97 (0.83–1.12)	0.658	0.94 (0.84–1.05)	0.259
Ischemic heart disease		1.01 (0.81–1.27)	0.912	1.00 (0.85–1.17)	0.993
Stroke		1.44 (1.11–1.88)	0.006	1.27 (1.06–1.53)	0.008
Heart failure		1.51 (1.11–2.06)	0.008	1.48 (1.21–1.80)	< 0.001
Chronic renal failure		0.85 (0.40–1.82)	0.673	0.85 (0.49–1.49)	0.578
Advanced liver disease		0.80 (0.39–1.65)	0.554	0.72 (0.41–1.28)	0.264
COPD		2.97 (2.17–4.06)	< 0.001	2.37 (1.97–2.85)	< 0.001
Malignancy		1.31 (0.98–1.74)	0.065	1.13 (0.92–1.38)	0.253

BMI: Basal mass index; COPD: Chronic obstructive pulmonary disease.

## Discussion

In this population-based cohort study of patients who were free from both ACS and pneumonia for at least two years, we observed a significantly higher incidence of pneumonia, with an IRR of 1.23, in the group with new exposure to ACS than in controls. The exposure to ACS was consistently an independent risk factor for pneumonia in three different survival models. To the best of our knowledge, this report provides the first evidence of reverse causality between pneumonia and ACS.

The heart and lung are closely-related vital organs, in terms of location and function, as they work as a team to oxygenate the cells and tissues of the body. Although the cross-relationship between these two organs has been extensively studied, researchers^11,16,26,29^ have focused on the fact that “acute respiratory infections can trigger acute cardiac events,” with inflammation, endothelial dysfunction, and platelet/thrombotic activation suggested to play important roles. However, considering the proposed mechanism, the reverse correlation can be suitably suspected, and this reverse causality should be fully considered.

Systemic inflammation^36,37^ has been considered to be a component of ACS, with the activation of circulating lymphocytes, monocytes, and neutrophils and cytokine responses; and the elevation of systemic inflammation markers, such as C-reactive protein^38,39^. Studies have shown that acute systemic cellular responses and the elevation of systemic inflammation markers in ACS are primary responses, rather than the byproducts of plaque rupture, thrombosis, or myocardial necrosis^36,40^. Therefore, the upregulated systemic inflammation in patients with ACS would render them susceptible to pneumonia.

Endothelial dysfunction has a pivotal role in all phases of atherosclerosis, from initiation to atherothrombotic complication^41^. As dysfunctional endothelium encourages the recruitment of leukocytes into the arterial wall and thereby predisposes to inflammation and plaque disruption in patients with ACS^42^. The accumulation of oxidative-damage products and failure to adapt to reactive oxygen species in ACS can result immune system activation and a proinflammatory milieu, generating functional and structural abnormalities, and consequently evoking cell death^43^. Cardiomyocytes are also able to promote distant organ damage following ischemic and mechanical injury via the innate immune system response, neurohormonal signaling, and, possibly, by the release of metabolic products (e.g., catalytic iron)^43^. Therefore, the lung is inevitably prone to infection in this situation because the lung is a highly immunologic organ, representing a gateway to the environment^43^.

Platelets have been suggested to contribute to diverse immunological processes, extending beyond the traditional role of hemostasis and thrombosis^44^. There is robust evidence that platelets play an active role in immunity as follows; intervention against microbial threats; recruitment and promotion of innate effector cell functions; modulation of antigen presentation; and enhancement of adaptive immune responses^45,46^. Clinical studies have shown that platelets might be important in the initial clearance of pathogens, as an increased risk of sepsis was observed among anti-platelet agent treated patients with community-acquired pneumonia. In a retrospective cohort study using the Medicaid database of 2013^45^, pneumonia incidence was significantly increased with clopidogrel exposure (OR 3.39, 95% CI 3.27–3.51, *p* < 0.0001), even after adjustment (aOR 1.48, 95% CI 1.41–1.55, *p* < 0.0001). Also, a nested cohort study showed that the use of aspirin in critically ill patients was associated with higher risk of ICU-acquired severe sepsis (aOR 1.70, 95% CI 1.08–2.70, *p* = 0.02), increased mechanical ventilation duration (aOR 2.7, 95% CI 0.51–4.90, *p* = 0.02) and ICU length of stay (OR 2.67, 95% CI 0.38–4.96, *p* = 0.02)^47^. Therefore, the use of anti-platelet agents in patients with ACS might play a role in the development of pneumonia via hindering the immune responses by platelet.

The present study has several strengths and important implications. First, the present study provides the first assessment of the impact of an ACS event on the incidence of pneumonia. Second, the present study used national claims data from representative community samples of adults, with a large sample size, and a comprehensive approach to identify pneumonia events (including both admission and outpatient settings). Furthermore, the results were replicated in multiple statistical models, with adjustment for a large number of potential confounders including smoking history. In addition to ascertaining the first event for pneumonia, we also accounted for the potential effect of recurrent pneumonia episodes and terminal events using the joint frailty model. Third, as we only included participants without pneumonia prior to ACS, our findings suggest that hospitalization for ACS should be considered as an independent risk factor of pneumonia in future strategies for the vaccination of pneumonia. This is particularly important in elderly individuals, as their risk of ACS and subsequent pneumonia is high.

The limitations of the present cohort study include those inherent to its retrospective design, the use of national claims data, and the approximation of clinical outcomes from an administrative database. The use of the coding system may not accurately reflect the endpoints, which may have affected the quantification of the effects of ACS observed in the present study sample. Additionally, we could not explore the severity of ACS and pneumonia in our cohort. However, most of the ACS diagnosis is relatively quite clear and straightforward, so the concern of the coding error might be minimized. Second, medical contact may be greater in patients with ACS than in other patients in the NHIS-HEALS; thus, the patients with ACS may have been more likely to be diagnosed with pneumonia and may have had a higher likelihood of being prescribed antibiotics earlier because of closer follow-up in the health care system. Third, although we adjusted for a large number of confounders, we could not assess the role of pneumococcal vaccination. Fourth, patients with ACS are more susceptible to acute pulmonary edema, which is sometimes difficult to differentiate from pneumonia. It is possible that the increased pneumonia incidence with ACS exposure, especially in the earlier period of observation, could be due to the misdiagnosis of pulmonary edema as pneumonia. However, we selected only pneumonia episodes with a total duration of antibiotic treatment of at least five days. Most patients with simple pulmonary edema, we do not use antibiotics for more than 5 days.

## Conclusion

We report that the hospitalization for ACS substantially increased the risk of pneumonia in a matched cohort of patients who were free from both ACS and pneumonia and were observed for up to 10 years. This information should be considered when formulating post-discharge care plans and preventive vaccination strategies in patients with ACS.

## Supplementary Information

Supplementary Information 1.

Supplementary Information 2.
