# Supplementary material for: Hospitalization for acute coronary syndrome increases the long-term risk of pneumonia: a population-based cohort study
Source: Sci Rep. 2021 May 6;11:9696. doi: 10.1038/s41598-021-89038-1 (PMC8102567; doi:10.1038/s41598-021-89038-1)
Supplement: Supplementary file 1 — Supplementary Information 1. [file 41598_2021_89038_MOESM1_ESM.docx]

**Title: Hospitalization for acute coronary syndrome increases the long-term risk of pneumonia: A population-based cohort study**

**Running title:** Risk of pneumonia after acute coronary syndrome

**Authors:** Joonghee Kim^1^, Sang Jun Park^2^, Sangbum Choi^3^, Won-woo Seo^4^, Yeon Joo Lee^5^

**Departments and Institutions**

^1^Department of Emergency Medicine, Seoul National University Bundang Hospital, Bundang-gu, Seongnam-si, Gyeonggi-do, Republic of Korea

^2^Department of Ophthalmology, Seoul National University Bundang Hospital, Bundang-gu, Seongnam-si, Gyeonggi-do, Republic of Korea

^3^Department of Statistics, Korea University, Seoul, Republic of Korea

^4^Division of Cardiology, Department of Internal Medicine, Kangdong Sacred Heart Hospital, Hallym University College of Medicine, Seoul, Republic of Korea

^5^Division of Pulmonary and Critical Care Medicine, Department of Internal Medicine, Seoul National University Bundang Hospital, Bundang-gu, Seongnam-si, Gyeonggi-do, Republic of Korea

**Corresponding author**: Yeon Joo Lee

Division of Pulmonary and Critical Care Medicine, Department of Internal Medicine, Seoul National University Bundang Hospital

82, Gumi-ro 173Beon-gil, Bundang-gu, Seongnam-si, Gyeonggi-do, 13620, Korea

Tel: 031-787-7082, Fax: 031-787-4052, E-mail:yjlee1117@snubh.org

Coding conventions for the diagnostic codes for comorbidities

1. Dot (.) is omitted from diagnostic codes (i.e. J38.3 🡪 J383)
2. If there are more than a code in the same hierarchy (i.e. J38 and J383), the parent codes indicate only themselves specifically, but not its children (i.e. J38x).
3. The code at the end of each of hierarchy, including those without any parent, indicate both themselves and their children (i.e. J37x for J37)
4. A comorbidity is coded as present if it has been stated as present explicitly in the latest health exam report regardless of the patient’s claim history.

Table S1. Operational definitions for comorbidities

|  | international Classification of Disease (ICD)-10 criteria | Scan range (past years) | | Minimum number of events | | Additional criteria |
| --- | --- | --- | --- | --- | --- | --- |
|  |  | Admission | Non-admission | Admission | Non-admission |  |
| Hypertension | I10, I11, I12, I13, I15 | 2 | 2 | 1 | 2 |  |
| Diabetes mellitus | E10, E11, E12, E13, E14 | 2 | 2 | 1 | 2 | Should be accompanied by oral hypoglycemic agent (ATC code: A10Bx) or insulin (A10Ax) |
| Chronic renal failure (including ESRD, below) | E1021, E1121, E1221, E1321, E1421, E1022, E1122, E1222, E1322, E1422, I120, I131, I132, N18, N19 | 2 | 2 | 1 | 2 | Also include end-stage renal disease or any kidney disability registration |
| - ESRD subset | N185, Z49, Z99, E1022, E1122, E1222, E1322, E1422 | 2 | 2 | 1 | 2 | Should be accompanied by renal replacement therapy or any patients with 1st-degree kidney disability registration |
| Ischemic heart disease | I20, I21, I22, I23, I24, I25 | 2 | 2 | 1 | 2 |  |
| Stroke | I60, I61, I62, I63,I64 | 2 | 2 | 1 | 2 |  |
| Heart failure | I50, I110, I255, I42, O903, I130, I132 | 2 | 2 | 1 | 2 |  |
| Advanced liver disease | I85, I864, I982, I983, K703, K704, K72, K746, K765, K766, K767, T864 | 2 | 2 | 1 | 2 |  |
| COPD | J43, J44 | 2 | 2 | 1 | 2 |  |
| Malignancy | C | 2 | 2 | 1 | 2 |  |

ATC: Anatomical Therapeutic Chemical Classification
