# Supplementary material for: Hospitalization for acute coronary syndrome increases the long-term risk of pneumonia: a population-based cohort study
Source: Sci Rep. 2021 May 6;11:9696. doi: 10.1038/s41598-021-89038-1 (PMC8102567; doi:10.1038/s41598-021-89038-1)
Supplement: Supplementary file 2 — Supplementary Information 2. [file 41598_2021_89038_MOESM2_ESM.docx]

**Title: Hospitalization for acute coronary syndrome increases the long-term risk of pneumonia: A population-based cohort study**

**Running title:** Risk of pneumonia after acute coronary syndrome

**Authors:** Joonghee Kim^1^, Sang Jun Park^2^, Sangbum Choi^3^, Won-woo Seo^4^, Yeon Joo Lee^5^

**Departments and Institutions**

^1^Department of Emergency Medicine, Seoul National University Bundang Hospital, Bundang-gu, Seongnam-si, Gyeonggi-do, Republic of Korea

^2^Department of Ophthalmology, Seoul National University Bundang Hospital, Bundang-gu, Seongnam-si, Gyeonggi-do, Republic of Korea

^3^Department of Statistics, Korea University, Seoul, Republic of Korea

^4^Division of Cardiology, Department of Internal Medicine, Kangdong Sacred Heart Hospital, Hallym University College of Medicine, Seoul, Republic of Korea

^5^Division of Pulmonary and Critical Care Medicine, Department of Internal Medicine, Seoul National University Bundang Hospital, Bundang-gu, Seongnam-si, Gyeonggi-do, Republic of Korea

**Corresponding author**: Yeon Joo Lee

Division of Pulmonary and Critical Care Medicine, Department of Internal Medicine, Seoul National University Bundang Hospital

82, Gumi-ro 173Beon-gil, Bundang-gu, Seongnam-si, Gyeonggi-do, 13620, Korea

Tel: 031-787-7082, Fax: 031-787-4052, E-mail:yjlee1117@snubh.org

Table S2. Sub-model for the terminal event (death) of the joint frailty model

|  |  | HR (95% CI) | p |
| --- | --- | --- | --- |
| Acute coronary syndrome | | 1.01 (0.90-1.14) | 0.823 |
| Age | 55 or less | Reference | |
|  | 56-65 | 0.98 (0.86-1.11) | 0.729 |
|  | 66-75 | 0.97 (0.85-1.12) | 0.711 |
|  | over 75 | 1.02 (0.85-1.22) | 0.837 |
| Smoking | Non-smoker | Reference | |
|  | Active smoker | 0.96 (0.84-1.09) | 0.512 |
|  | Ex-smoker | 1.03 (0.89-1.19) | 0.682 |
| BMI | Underweight (<18.5) | 1.01 (0.75-1.38) | 0.925 |
|  | Normal (18.5 – 22.9) | Reference | |
|  | Overweight (23.0 – 24.9) | 1.01 (0.89-1.14) | 0.896 |
|  | Pre-Obese (25.0 – 29.9) | 1.00 (0.88-1.12) | 0.937 |
|  | Obese (>=30) | 0.90 (0.65-1.23) | 0.499 |
| Sex, male |  | 1.06 (0.94-1.20) | 0.343 |
| Diabetes |  | 1.03 (0.90-1.18) | 0.655 |
| Hypertension | | 0.92 (0.82-1.02) | 0.115 |
| Ischemic heart disease | | 1.06 (0.90-1.25) | 0.495 |
| Stroke |  | 1.23 (1.00-1.51) | 0.045 |
| Heart failure | | 1.00 (0.78-1.27) | 0.970 |
| Chronic renal failure | | 0.76 (0.42-1.36) | 0.357 |
| Advanced liver disease | | 0.76 (0.44-1.31) | 0.329 |
| COPD |  | 0.96 (0.72-1.27) | 0.778 |
| Malignancy |  | 1.08 (0.88-1.34) | 0.458 |

BMI: Basal mass index; COPD: chronic obstructive pulmonary disease
